# One Health approach to radionuclides in milk and meat: Transfer mechanisms, processing effects, regulatory limits, and mitigation strategies

**DOI:** 10.14202/vetworld.2026.3568-3594

**Published:** 2026-08-17

**Authors:** Abdykarimova Shynar, Serikova Ainur, Abzal Kereyev, Duyssembaev Sergazy, Amirgalina Zhadyra, Koigeldinova Ainur, Suleimenov Shyngys, Akhmadiyeva Ainur, Balgabaikyzy Assem, Serikov Zhaxylyk, Paritova Assel, Valiyeva Zhadyra

**Affiliations:** 1 Shakarim University, Semey 071412, Republic of Kazakhstan.; 2 West Kazakhstan Agrarian and Technical University named after Zhangir Khan, Uralsk 090009, Republic of Kazakhstan.; 3 Saken Seifullin Kazakh Agro-Technical Research University, Astana 010011, Republic of Kazakhstan.

**Keywords:** animal products, food processing, milk, One Health, radiological safety, radionuclides, transfer mechanisms, veterinary public health

## INTRODUCTION

Radionuclides in milk and meat constitute an important food safety and public health concern because of persistent environmental contamination arising from nuclear accidents, nuclear weapons testing, and naturally occurring radioactive material (NORM) [1]. Since the mid-20th century, radionuclides such as ¹³⁷Cs and ⁹⁰Sr have been shown to transfer through terrestrial food chains and accumulate in animal-derived foods, thereby contributing to human internal radiation exposure [2]. The Chernobyl and Fukushima nuclear accidents substantially expanded global surveillance programs and improved understanding of radionuclide transfer, long term environmental persistence, and contamination pathways, leading to the development of regulatory frameworks for food safety worldwide [3]. Because milk and meat are widely consumed dietary staples, understanding radionuclide transfer, distribution, and persistence in these products is essential for radiation protection, veterinary public health, food safety, and sustainable livestock production [4]. Although long term monitoring programs in Japan, Europe, and other regions indicate that radionuclide concentrations in most commercial milk and meat remain below regulatory limits, localized contamination persists in certain geographical regions, production systems, and food categories [1].

Radionuclide transfer to animal-derived foods is influenced by complex interactions among environmental contamination, soil characteristics, feed composition, animal physiology, production systems, food processing, and regulatory interventions [5]. Despite considerable research, several important knowledge gaps remain. Existing studies are highly heterogeneous with respect to radionuclides investigated, livestock species, production systems, and analytical methodologies, limiting direct comparison among studies and reducing the applicability of generalized transfer parameters. Uncertainties also remain regarding interspecies variation in transfer coefficients, radionuclide-specific behavior, the relative contributions of soil-to-plant and feed-to-animal transfer pathways, and the effectiveness of mitigation strategies under different environmental and management conditions [6]. Furthermore, most published reviews have focused on individual aspects of radionuclide contamination, such as environmental transfer, dose assessment, or regulatory monitoring, whereas comprehensive integration of transfer mechanisms, food processing, regulatory frameworks, monitoring systems, and mitigation strategies remains limited. The available evidence is also geographically imbalanced, with studies from temperate and post-accident regions greatly outnumbering those from tropical, monsoonal, and developing production systems. Similarly, dairy and beef cattle have received substantially greater research attention than sheep, goats, buffaloes, camels, poultry, and aquaculture species. In addition, radiological risk extends beyond nuclear power plant accidents to legacy contamination from weapons testing, including the Semipalatinsk Test Site [7], and exposure to NORM [8, 9]. These limitations highlight the need for an updated and integrated review that synthesizes recent evidence within a One Health framework encompassing environmental, animal, food, and human health.

This review synthesizes current evidence on the mechanisms governing radionuclide transfer to milk and meat, evaluates the influence of food processing on radionuclide distribution, examines international regulatory standards and monitoring approaches, and summarizes evidence-based mitigation strategies for reducing radionuclide contamination throughout the food chain. Particular emphasis is placed on literature published between 2020 and 2026 to provide an updated overview of recent scientific advances. The review integrates environmental transfer processes, animal physiological factors, food processing redistribution through processing factor (Pf) and retention factor (Fr), regulatory frameworks, monitoring strategies, and farm-to-consumer mitigation measures within a comprehensive One Health perspective. In addition, emerging factors influencing radionuclide transfer, including gut microbiota, chronobiological variation, diverse production systems, and climate-related environmental conditions, are discussed to identify current knowledge gaps and future research priorities. By consolidating recent evidence across these interconnected disciplines, this review aims to support improved risk assessment, radiological surveillance, food safety management, and policy development for the protection of animal and human health.

## REVIEW METHODOLOGY

### Search strategy and literature identification

This review followed the Preferred Reporting Items for Systematic Reviews and Meta-Analyses (PRISMA) 2020 guidelines, adapted for narrative synthesis. Four electronic databases, PubMed, Scopus, Web of Science, and Google Scholar, were searched. The database search was supplemented by searches of regulatory repositories, including the International Atomic Energy Agency (IAEA), Codex Alimentarius, the World Health Organization, relevant national agencies, and manual screening of reference lists. Peer-reviewed articles published between January 2020 and May 2026 were prioritized. However, seminal sources published before 2020 were retained when they remained the primary or authoritative references for foundational transfer parameters, post-Chernobyl countermeasure data from 1988 to 1998, physical constants, and current regulatory instruments. The search terms combined keywords related to radionuclides, animal products, transfer mechanisms, processing, and countermeasures using Boolean operators. No language restrictions were applied.

### Inclusion and exclusion criteria

Studies were eligible for inclusion if they reported original empirical data or modeling of radionuclide transfer to milk or meat from livestock species, including cattle, goats, sheep, poultry, horses, and aquaculture species, and addressed at least one core domain: environmental transfer, animal physiology, food processing, regulation, monitoring, or countermeasures. Records were excluded if they did not contain data on animal-derived products, were abstracts without accessible full texts, were non-empirical commentaries, or lacked sufficient methodological detail to support reproducibility.

### Study selection

Scientific articles, technical reports, and regulatory documents identified through the literature search were compiled, and duplicate records were removed before screening. Titles and abstracts were initially screened against the predefined eligibility criteria. The full texts of potentially relevant articles and reports were subsequently assessed, and records meeting the eligibility criteria were included in the narrative synthesis. Because this was a narrative review conducted using a structured, PRISMA-informed search rather than a formal systematic review, stage-specific screening counts were not tabulated. A total of 124 studies and reports met the inclusion criteria and were included in the final synthesis.

### Data extraction and quality assessment

A standardized data extraction form was used to record the publication year, geographical region, radionuclide(s), animal species, production system, transfer parameters (Fm, Ff, concentration ratio (CR), and aggregated transfer coefficient [Tag]), processing factors (Pf and Fr), regulatory limits, and countermeasure effectiveness. Study quality was assessed qualitatively based on the clarity of the methodology, sample size, validation of the limit of detection (LOD) and limit of quantification (LOQ), treatment of uncertainty, and relevance to real-world conditions. Studies with stronger methodological quality and greater practical relevance were prioritized during the synthesis. Formal validated quality assessment instruments, such as those developed by the Joanna Briggs Institute (JBI) and the Office of Health Assessment and Translation (OHAT), were not applied, consistent with the narrative design of the review.

### Data synthesis and analysis

**Figure 1:**
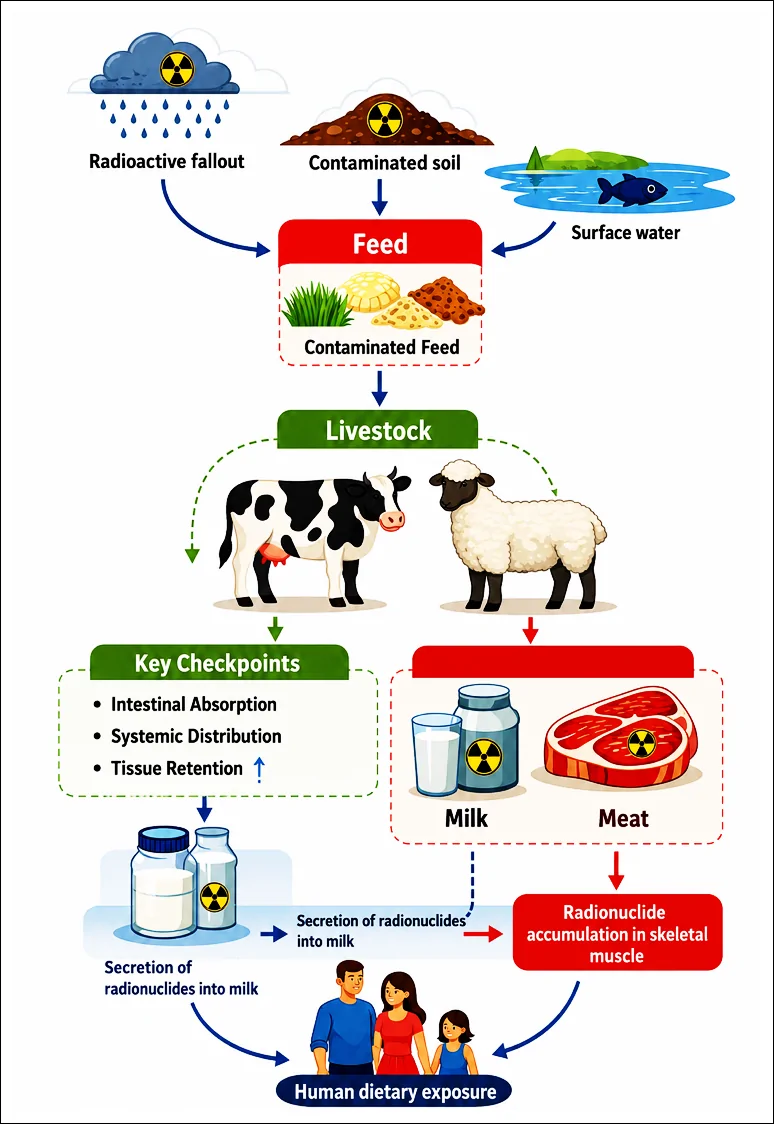
Conceptual framework illustrating radionuclide transfer from the environment to humans through livestock products [26].

**Figure 2:**
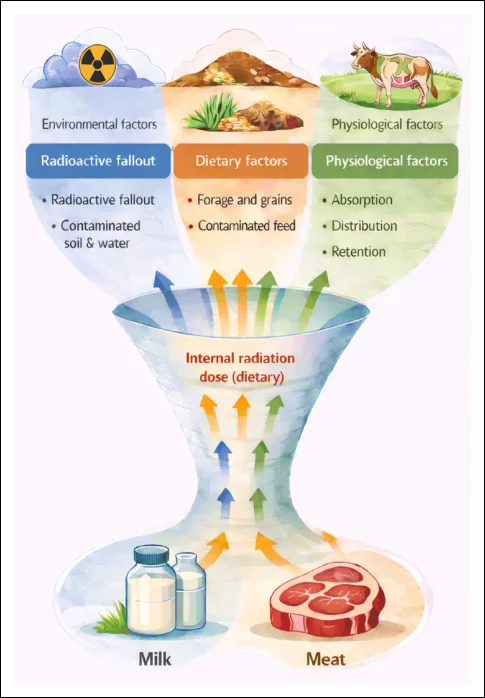
Conceptual tornado diagram illustrating the relative influence of environmental, dietary, and physiological factors on radionuclide transfer to milk and meat.

Because of substantial heterogeneity in study designs, radionuclides, animal species, production systems, analytical methods, and reporting approaches, a meta-analysis was not considered feasible. Therefore, the evidence was synthesized narratively and supported by tabular and graphical summaries. Quantitative transfer parameters were compiled in tables and reported as ranges and, where available, geometric means. The conceptual frameworks presented in Figures 1 and 2 integrate environmental, physiological, processing, and regulatory pathways within a One Health framework.

**MECHANISMS:** Biochemical and physiological determinants of radionuclide accumulation

### Radionuclide-specific biological behavior

Radionuclide behavior in livestock is primarily determined by the biochemical mimicry of essential elements, which governs their absorption, distribution, retention, and excretion. This review updates conventional analog-based classifications by incorporating recent livestock-specific evidence from broilers, horses, and cattle, while also highlighting the limited understanding of co-contaminant interactions and their potential influence on radionuclide transfer.

¹³⁷Cs behaves as a potassium analog and is distributed predominantly throughout soft tissues, making skeletal muscle the principal edible reservoir. In cattle, the transfer of ¹³⁷Cs from feed is greater to meat than to milk, with equilibrium transfer coefficients of 0.0062 d kg^-1 and 0.0022 d L^-1, respectively. Approximately 5.7–7.4% of the daily dietary intake of ¹³⁷Cs is transferred to milk, demonstrating its importance in both meat and dairy production systems [10, 11].

In contrast, ⁹⁰Sr behaves as a calcium (Ca) analog and accumulates preferentially in mineralized tissues, particularly bone. Consequently, only 0.02–0.06% of the daily intake is transferred to muscle, whereas 5.47–6.47% is retained in skeletal tissues, reflecting its strong affinity for the mineral component of bone and its long term persistence within the animal [10].

¹³¹I follows the normal metabolic pathway of stable iodine and is efficiently absorbed and secreted into milk, with an equilibrium transfer coefficient of 0.009 d L^-1, which is greater than that reported for ¹³⁷Cs. This rapid transfer pathway resulted in milk becoming the principal source of human exposure to ¹³¹I following the Chernobyl nuclear accident. However, because of its relatively short physical half-life, radioiodine is primarily considered an early-phase radiological hazard [11–13].

In contrast, the actinides ²⁴¹Am and ²³⁹Pu/²⁴⁰Pu exhibit extremely low transfer to edible animal products. These radionuclides accumulate predominantly in the liver and, to a lesser extent, mineral-associated tissues, resulting in substantially lower contamination of milk and meat than that observed for ¹³⁷Cs and ⁹⁰Sr [14, 15].

The principal biological characteristics, target organs, and transfer patterns of the major fallout radionuclides associated with milk and meat are summarized in Table 1, based on published experimental and field studies involving livestock and other animal models [10–14].

**Table 1:** Comparative behavior of key fallout radionuclides in milk and meat.

**Radionuclide**	**Main biological ** **analog** **/target**	**Behavior** ** in animals, milk, and meat**	**Typical transfer/levels**
¹³⁷Cs	Potassium analog; distributed predominantly in soft tissues, especially skeletal muscle	Relatively uniform whole-body distribution; efficient accumulation in muscle and moderate transfer to milk; remains a major concern during long term forage contamination	Cattle: Milk transfer coefficient = 0.0022 d L^-1; meat transfer coefficient = 0.0062 d kg^-1; daily transfer to milk = 5.72–7.36%; muscle transfer from ration = 2.0–6.37%; representative muscle activity concentrations and milk concentrations = 0.13–4.07 Bq L^-1 in surveyed production systems [10, 11]
⁹⁰Sr	Calcium analog; preferential accumulation in bone	Selective accumulation in skeletal tissues; low transfer to muscle and lower transfer to milk than ¹³⁷Cs; remains important because of long term persistence and skeletal retention	Cattle: Milk transfer = 1.18–1.65% of daily intake; muscle transfer = 0.02–0.06%; bone retention = 5.47–6.47%; representative activity concentrations: bone = 0.61–12.36 Bq kg^-1, muscle = <0.01–0.14 Bq kg^-1, milk = 0.02–1.36 Bq L^-1 [10]
¹³¹I	Stable iodine analog; concentrated in the thyroid gland and rapidly transferred to milk	Rapid absorption and secretion into milk; principal short-term radionuclide in dairy food chains following nuclear releases; major contributor to thyroid radiation dose in humans	Cattle: Equilibrium milk transfer coefficient = 0.009 d L^-1 for iodide, exceeding that of ¹³⁷Cs; approximately 11,000 milk samples analyzed following the Chernobyl accident [11–13]
²⁴¹Am	Actinide; retained primarily in the liver and mineral-associated tissues	Liver is the principal site of accumulation; food chain significance is substantially lower than that of Cs and strontium radionuclides	Horse study: Maximum TF = 72 ± 22 × 10^-5 d kg^-1 FW in the liver following ingestion of leachate-contaminated feed; lower transfer observed following soil ingestion [14]
²³⁹Pu/²⁴⁰Pu	Actinides; retained primarily in the liver and bone-associated tissues	Extremely limited transfer to edible soft tissues; tissue transfer substantially lower than that of ¹³⁷Cs and ⁹⁰Sr under comparable feeding conditions	Horse study: Maximum TF = 31.8 ± 8 × 10^-5 d kg^-1 FW in the liver; transfer following soil ingestion was up to three orders of magnitude lower than that following ingestion of soluble leachate-contaminated feed [14]

Am = Americium; Bq = Becquerel; CR = Concentration ratio; Cs = Cesium; Fm = Transfer coefficient to meat; Fr = Retention factor; FW = Fresh weight; I = Iodine; Pf = Processing factor; Pu = Plutonium; Sr = Strontium; TF = Transfer factor.

### Physiological modifiers of radionuclide transfer

Radionuclide transfer in livestock reflects the interplay of physiological, dietary, and source-dependent processes rather than fixed feed-to-product coefficients. Experimental chronobiological evidence demonstrates circadian modulation of tissue retention. Administration of ¹³¹I to mice at midday increased radionuclide retention by approximately 9% in the thyroid and 30%–50% in the salivary glands and intestine compared with late-day administration [16], highlighting temporal uncertainty that is rarely incorporated into transfer models. Age and growth also modify radionuclide kinetics. Young animals exhibit greater uptake per unit of intake, particularly for bone-seeking Sr; however, growth dilution may shorten effective half-lives [5, 17, 18].

Transfer to milk depends on gastrointestinal absorption, milk yield, and dry matter intake [4, 19], with marked variation between high-yielding temperate breeds and lower-yielding tropical production systems [18]. Dietary composition and mineral status provide additional mechanistic modulation. Ca suppresses Sr absorption, whereas forage-to-concentrate ratios and feed digestibility influence Cs bioavailability and endogenous excretion [20]. Soil–plant chemistry and farm management practices further contribute to variability in radionuclide transfer [21, 22].

Emerging evidence indicates that radiation exposure and changes in the gut microbiota may alter epithelial integrity and mineral absorption [23, 24]; however, livestock-specific evidence remains limited. Overall, important sources of uncertainty include physiological state, breed, diet, and the bioavailability of radionuclides from different sources.

These observations support a multiscale framework for radionuclide transfer that links molecular processes, including competition involving ion channels and nutrient transporters and gut microbiota-mediated modulation of mineral absorption, with organ-level kinetics, such as chronobiological variation in retention, and whole-animal factors, including breed, age, growth dilution, milk yield, and diet. Chronobiology and the gut microbiome are rarely incorporated into conventional transfer models, although both may substantially influence radionuclide retention and absorption. Their integration could improve the accuracy of dose models under changing climatic conditions and feeding regimes. Recognizing these modifiers as an emerging research frontier extends earlier analog- and coefficient-based descriptions of radionuclide transfer.

### Tissue- and fraction-specific distribution

Radionuclide distribution in animal products is strongly tissue- and fraction-specific, challenging the use of a single transfer coefficient. In wildlife inhabiting nuclear test areas, ¹³⁷Cs preferentially accumulated in skeletal muscle, followed by the kidneys and liver, whereas ⁹⁰Sr accumulated primarily in bone [7, 25]. These patterns are consistent with the experimentally established potassium analog behavior of Cs and Ca-analog behavior of Sr [19]. Controlled studies in broiler chickens have further demonstrated element- and tissue-specific equilibrium times and transfer factors (TF) for ¹³⁷Cs and ²⁴¹Am [20], indicating multicomponent kinetics rather than uniform whole-body burdens. However, extrapolation from wild ungulates to livestock remains uncertain because of differences in diet, growth rate, physiological status, and bone turnover.

In milk, radionuclide partitioning is governed by physicochemical speciation. Casein micelles bind most Zn and substantial proportions of Sr analogs, fat globules retain smaller but potentially relevant fractions, and the aqueous phase contains Li, B, Rb, and other soluble elements [26]. By analogy, Cs, which behaves similarly to K, and Sr, which behaves similarly to Ca, are distributed among the whey, casein, and fat fractions according to their ionic properties [6, 27]. This distribution influences radionuclide losses during processing and the resulting consumer dose. Nevertheless, quantitative fractionation data remain limited for many radionuclides, particularly across different breeds and production systems.

Cheesemaking promotes the partitioning of radiocesium (r-Cs) into whey, thereby reducing the concentration of ¹³⁷Cs in casein-based dairy products. In contrast, ⁹⁰Sr is associated with Ca metabolism and is retained primarily in the curd, whereas lipophilic natural radionuclides may become concentrated in milk fat [6].

Because fractionation continues after radionuclides leave the animal, the farm-to-fork framework was extended to include processing fractionation as a control point (Figure 1). Food processing, therefore, represents a manageable One Health intervention point that links environmental contamination and animal physiology to consumer exposure [26].

Radionuclides released into the environment through atmospheric deposition and the contamination of soil and surface water enter livestock production systems primarily through contaminated feed and drinking water. Following ingestion, radionuclides undergo gastrointestinal absorption, systemic distribution, and tissue retention governed by physiological and biochemical processes. A proportion may be rapidly secreted into milk through the mammary gland, whereas longer-term retention may occur in skeletal muscle and other tissues, contributing to contamination of meat and edible organs.

The consumption of contaminated milk and meat therefore represents an important dietary pathway of human exposure to radionuclides.

**TRANSPORT:** Feed-to-product transfer and modeling approaches

### Definitions and transfer metrics

Quantitative assessment of radionuclide transfer in livestock production systems relies primarily on feed-to-product transfer coefficients, including Fm for feed-to-milk transfer (d·L⁻¹) and Ff for feed-to-meat transfer (d·kg⁻¹), which are typically calculated under steady-state assumptions. Field data demonstrate substantial variability in these parameters. In tropical monsoon production systems, the Fm of stable Sr ranged from 2.2 × 10⁻³ to 7.2 × 10⁻³ d·L⁻¹, with a site mean of 3.2 × 10⁻³ d·L⁻¹ [18]. In Ugandan grazing systems, plant-to-milk transfer ratios for naturally occurring U and Th ranged from 0.05 to 0.17 [28]. These measurements differ from the conventions described in IAEA Technical Reports Series No. 472, which distinguishes Fm from simpler CR. Unlike Fm, CRs do not incorporate radionuclide intake and may therefore obscure important dietary determinants of transfer [18, 29].

For meat, Ff varies according to the radionuclide, tissue, animal species, and exposure pathway. In broiler chickens, the Ff of ¹³⁷Cs was 1.9 ± 0.3 d·kg⁻¹ for the forage-to-muscle pathway and 0.18 ± 0.05 d·kg⁻¹ for the soil-to-muscle pathway, whereas the soil-to-muscle Ff of ²⁴¹Am was 7.5 × 10⁻⁵ d·kg⁻¹ [20]. These findings highlight the strong influence of source-dependent bioavailability. In horses, the transfer of ²³⁹⁺²⁴⁰Pu, ²⁴¹Am, ¹³⁷Cs, and ⁹⁰Sr differed by several orders of magnitude among muscle, liver, and bone [14]. Reported transfer parameters frequently span three to four orders of magnitude due to differences among animal species, diets, productivity, soil chemistry, radionuclide speciation, and analytical methodologies. Representative Fm, Ff, and TF values for selected radionuclides, animal species, products, and exposure pathways are summarized in Table 2[14, 18, 20, 28].

**Table 2:** Illustrative Fm, Ff, and TF values for selected radionuclides and animal species.

**Pathway and radionuclide**	**Parameter and typical magnitude**	**Notes**
Grass → cow milk, stable Sr	Fm ≈ 2.2 × 10⁻³–7.2 × 10⁻³ d·L⁻¹; site mean = 3.2 × 10⁻³ d·L⁻¹	Tropical monsoon production system involving field-grazing cows [18]
Pasture → cow milk, natural U and Th	Transfer ratio: U = 0.07–0.17; Th = 0.05–0.17	Ugandan grazing system; values used for dose estimation [28]
Grass meal → broiler muscle, ¹³⁷Cs	Ff ≈ 1.9 ± 0.3 d·kg⁻¹	Long term feeding with grass meal [20]
Soil → broiler muscle, ¹³⁷Cs	Ff ≈ 0.18 ± 0.05 d·kg⁻¹	Lower transfer from soil than from forage [20]
Soil → broiler muscle, ²⁴¹Am	Ff ≈ 7.5 × 10⁻⁵ d·kg⁻¹	Very low uptake by skeletal muscle [20]
Feed leachate → horse liver, ²⁴¹Am	Maximum TF = 72 ± 22 × 10⁻⁵ d·kg⁻¹ FW	Preferential accumulation in the liver [14]
Feed leachate → horse ribs, ⁹⁰Sr	Maximum TF = 720 ± 144 × 10⁻³ d·kg⁻¹ FW	The highest observed TF occurred in the ribs [14]

Abbreviations = Ff = Feed-to-meat transfer coefficient; Fm = Feed-to-milk transfer coefficient; FW = Fresh weight; TF = Transfer factor; Th = Thorium; U = Uranium.

Important controversies concern the validity of steady-state assumptions, the use of stable-element analogs, and the substitution of transfer coefficients among animal species and production systems. Uncertainty is further amplified when CRs are applied without corresponding feed intake data. Table 3 consolidates reported transfer parameters across radionuclides and animal species, including geometric means and measures of dispersion where available. The compiled values demonstrate the approximately lognormal and multi-order-of-magnitude variability of Fm, Ff, and TF, which limits the reliability of single generic transfer values [7, 9, 14, 18, 20, 21, 28, 30–37].

### CR, Tag, and Fm/Ff in radionuclide transfer

The concentration ratio (CR) and Tag are standard radioecological metrics used to quantify radionuclide transfer to milk and meat. CR is defined as the equilibrium ratio of radionuclide activity in an animal product to that in feed [18]. Because CR does not require explicit estimation of daily dry matter intake, it is readily applicable under field conditions [4]. However, its apparent stability across species depends on near-steady-state exposure and relatively consistent diets. Uncertainty therefore increases during transient contamination events and seasonal changes in feed composition [4, 18].

**Table 3:** Consolidated feed-to-product transfer parameters for radionuclides in milk and meat across animal species, including central tendency and dispersion.

**Radionuclide**	**System, species, product, ** **and pathway**	**Parameter and central value**	**Range or dispersion**	**Reference**
¹³⁷Cs	Cow milk, grass → milk	Fm; GM ≈ 2.22 × 10⁻² d·L⁻¹	1.0 × 10⁻²–3.71 × 10⁻² d·L⁻¹	[30]
¹³⁷Cs	Cow milk, feed → milk	Reference Fm ≈ 3.0 × 10⁻³ d·L⁻¹	5.4 ± 0.5 × 10⁻³ d·L⁻¹; lognormal distribution	[31, 32]
¹³⁷Cs	Cow and sheep milk	Sheep milk Fm ≈ 0.06 d·L⁻¹; cow milk Fm ≈ 0.003 d·L⁻¹	Sheep milk value approximately 20-fold greater than the cow milk value	[32]
¹³⁷Cs	Cattle, sheep, and poultry meat, feed → muscle	Ff: beef = 0.01 d·kg⁻¹; veal = 0.35 d·kg⁻¹; sheep = 0.33 d·kg⁻¹; chicken = 1.3 d·kg⁻¹	Values span approximately two orders of magnitude among species	[32]
¹³⁷Cs	Broiler muscle, forage → muscle and soil → muscle	Ff = 1.9 ± 0.3 d·kg⁻¹ for forage; 0.18 ± 0.05 d·kg⁻¹ for soil	Strongly dependent on the contamination source	[20]
²⁴¹Am	Broiler muscle, soil → meat	Ff = 7.5 × 10⁻⁵ d·kg⁻¹	Accumulation occurred predominantly in the liver and bone rather than in muscle	[20]
⁹⁰Sr	Cow milk, stable-Sr analog	Site GM Fm = 3.2 × 10⁻³ d·L⁻¹	Fm = 2.2 × 10⁻³–7.2 × 10⁻³ d·L⁻¹; soil-to-grass Fv GM = 1.8, with a range of 0.18–8.6	[18]
⁹⁰Sr	Horse ribs and bone	Maximum TF = 720 ± 144 × 10⁻³ d·kg⁻¹ FW	Strong affinity for bone	[14]
²⁴¹Am	Horse liver	Maximum TF = 72 ± 22 × 10⁻⁵ d·kg⁻¹ FW	Preferential hepatic accumulation	[14]
¹³¹I	Cow milk	Mean Fm = 6.7 ± 8.7 × 10⁻³ d·L⁻¹; median Fm = 4.0 × 10⁻³ d·L⁻¹	Equilibrium values = 5.6 × 10⁻³ and 6.3 × 10⁻³ d·L⁻¹	[33, 34]
¹³¹I	Goat and cow milk	Goat milk Fm ≈ 2.8 × 10⁻² d·L⁻¹; cow milk Fm ≈ 3.6 × 10⁻³ d·L⁻¹	Goat milk value approximately eightfold greater than the cow milk value	[35, 36]
¹³¹I	Sheep milk	Fm ≈ 0.29 ± 0.017 d·L⁻¹; approximately 56% ± 0.035% of intake secreted	Approximately one order of magnitude greater than values reported for cattle	[37]
²³⁹⁺²⁴⁰Pu and ²⁴¹Am	Cow, sheep, and goat milk, modeled using STS	≤6.5 × 10⁻² Bq·L⁻¹ for sheep and goats; ≤2.6 × 10⁻² Bq·L⁻¹ for cows	Model-derived values indicating very low activity concentrations	[7]
U and Th	Cow milk, pasture → milk	Transfer ratio = 0.05–0.17	Estimated dose was approximately 1.5% of the applicable limit	[28]
⁴⁰K and ²¹⁰Pb	Grass → cow milk and milk	Transfer parameter = 2 × 10⁻³–4 × 10⁻³	Measurements obtained from a phosphate-producing area	[9]
²³²Th, ²²⁶Ra, and ⁴⁰K	Cow milk, grass → milk	TF = 0.45, 0.166, and 0.11 d·L⁻¹, respectively	Values were below the corresponding global averages	[21]

Abbreviations = Bq = Becquerel; CR = Concentration ratio; Ff = Feed-to-meat transfer coefficient; Fm = Feed-to-milk transfer coefficient; Fv = Soil-to-vegetation transfer factor; FW = Fresh weight; GM = Geometric mean; STS = Standardized transfer scenario; TF = Transfer factor; Th = Thorium; U = Uranium.

Tag relates radionuclide deposition density, expressed as Bq·m⁻², directly to radionuclide activity in an animal product. This metric is particularly useful in extensive livestock and wildlife systems in which feed intake cannot be reliably quantified [4, 38]. Although Tag is operationally useful for emergency assessments, it integrates multiple processes along the soil–plant–animal pathway. Consequently, Tag may exhibit substantial variability among production systems and provides less mechanistic information than intake-based transfer coefficients.

Classical Fm and Ff coefficients relate radionuclide activity in milk and meat, respectively, to the daily radionuclide intake of the animal [4, 7]. No single transfer metric is universally superior, as the appropriate metric depends on the exposure scenario, the availability of intake data, and the intended application. The definitions, advantages, limitations, and preferred uses of CR, Tag, Fm, and Ff are summarized in Table 4.

### Radionuclide half-lives in milk and meat

Biological half-life (T₁/₂,biol), ecological half-life (T₁/₂,ecol), and effective half-life (T₁/₂,eff) determine the persistence of radionuclides in animal products. T₁/₂,biol describes radionuclide elimination through metabolic and physiological processes, excluding physical radioactive decay. T₁/₂,ecol represents the decline in environmental radionuclide activity resulting from migration, dilution, changes in bioavailability, and the implementation of countermeasures. T₁/₂,eff integrates environmental decline and physical radioactive decay according to the following relationship: 1/T₁/₂,eff = 1/T₁/₂,phys + 1/T₁/₂,ecol [39].

**Table 4:** Comparison of radionuclide transfer metrics for milk and meat, including their definitions, advantages, limitations, and preferred conditions of use.

**Metric**	**Definition**	**Advantages**	**Limitations**	**Preferred use**
CR	Equilibrium ratio of radionuclide activity in an animal product to that in feed; does not explicitly incorporate daily dry matter intake	Applicable under field conditions; does not require intake data; may show apparent stability across species	Valid primarily under near-steady-state conditions and consistent feeding; uncertainty increases during transient contamination and seasonal changes in feed	Routine monitoring under stable dietary and equilibrium conditions
Tag	Coefficient relating radionuclide deposition density, expressed as Bq·m⁻², directly to radionuclide activity in an animal product	Operationally useful for emergency assessment; applicable when feed intake cannot be quantified, including extensive livestock and wildlife systems	Integrates multiple soil–plant–animal processes, resulting in high cross-system variability and limited mechanistic transparency	Post-accident assessment, emergency response, and evaluation of extensive livestock or wildlife systems
Fm and Ff	Ratios of radionuclide activity in milk or meat to daily radionuclide intake; Fm applies to milk and Ff applies to meat	Mechanistically explicit; directly links intake with product contamination; suitable for dose modeling	Requires reliable intake data; reported values commonly span several orders of magnitude and may follow lognormal distributions, limiting the use of generic coefficients	Mechanistic modeling and dose assessment when radionuclide intake is known

Abbreviations = Bq = Becquerel; CR = Concentration ratio; Ff = Feed-to-meat transfer coefficient; Fm = Feed-to-milk transfer coefficient; Tag = Aggregated transfer coefficient.

Reported T₁/₂,biol values range from approximately 0.6 to 3.5 d in milk, with approximately 2 d commonly reported, and from 3 to 300 d in muscle, with approximately 30 d considered typical [19, 40]. These broad ranges reflect multicompartment radionuclide kinetics and substantial differences among animal species, physiological states, diets, exposure durations, and metabolic turnover. Considerable variation among production and environmental systems remains, particularly for Cs and Sr. Representative biological, ecological, effective, and physical half-lives of the principal radionuclides detected in milk and meat are summarized in Table 5 based on experimental studies and long term environmental monitoring data [19, 39–44].

**Table 5:** Biological, ecological, effective, and physical half-lives of selected radionuclides in milk, meat, and animal tissues

**Radionuclide**	**Compartment ** **or product**	**Biological half-life**	**Ecological, effective, or physical half-life**	**Reference**
¹³⁷Cs	Cow milk	Approximately 2 d; reported range = 0.6–3.5 d	Effective half-life ≈ 9 y in milk based on 25-y pasture monitoring; physical half-life ≈ 30 y	[19, 40, 41]
¹³⁷Cs	Muscle and meat	Approximately 30 d; reported range = 3–300 d	Ecological half-life = 8.0 y in chickens; effective half-life = 2.6–7.3 y in wild boars; physical half-life ≈ 30 y	[19, 39, 42]
⁹⁰Sr	Bone	Long biological persistence because of bone-seeking behavior	Slow ecological and biological turnover; physical half-life ≈ 29 y	[19, 43]
⁹⁰Sr	Milk	Several days in the soluble fraction	Reported ecological or effective half-life = 308 ± 57 d; physical half-life ≈ 10,410 d	[19, 44]

Abbreviations = d = Day; T₁/₂,biol = Biological half-life; T₁/₂,ecol = Ecological half-life; T₁/₂,eff = Effective half-life; T₁/₂,phys = Physical half-life; y = Year.

### Production systems and environmental context

Comparative evidence consistently demonstrates that pasture-based production systems result in greater and more rapid radionuclide transfer to milk and meat than controlled indoor feeding systems, although substantial variability exists among studies. Reported soil-to-forage TF and forage-to-milk Fm values span several orders of magnitude [18, 21, 22], reflecting differences in soil chemistry, plant species, climatic conditions, and management practices. Although transfer of naturally occurring U and Th is measurable, the resulting radiation doses generally remain approximately 1.5% of established safety limits [28, 45]. In contrast, ¹³⁷Cs and ⁹⁰Sr exhibit greater persistence and considerably higher variability among contaminated regions [20, 46]. These differences highlight the strong dependence of radionuclide transfer on environmental conditions and limit the application of generic transfer coefficients across production systems.

Indoor production systems generally reduce radionuclide intake through the use of selected and stored feeds [18, 19]. Lower ¹³⁷Cs and ⁹⁰Sr concentrations have been reported in stored feeds than in freshly grazed forage, although Fm values for Sr may remain comparable with those observed under pasture conditions when similar diets are provided [5]. Nevertheless, uncertainty remains due to temporal variations in radionuclide activity, heterogeneous feed sources, and incomplete information on feed intake.

Silvopastoral systems remain poorly characterized with respect to radionuclide transfer [47]. However, higher soil organic matter content and deeper rooting systems may reduce Cs bioavailability by enhancing radionuclide retention within the soil profile [48]. Across all production systems, soil characteristics, including clay content, organic matter, and exchangeable K, exert a major influence on radionuclide bioavailability. Clay minerals and humic substances reduce the mobility of Cs and Ra through adsorption and complex formation [49, 50], whereas elevated concentrations of exchangeable K suppress Cs uptake by plants and may influence radionuclide desorption dynamics [48, 51].

Two production systems represent important priorities for future investigation. First, silvopastoral systems integrating trees with pasture may reduce r-Cs bioavailability; however, quantitative transfer parameters for milk and meat remain largely unavailable [47, 48]. Second, aquaculture studies have shown that providing uncontaminated feed reduces ¹³⁷Cs and ⁹⁰Sr concentrations in fish, although ⁹⁰Sr declines more slowly because of continued uptake from water and preferential accumulation in bone [52]. Recent market surveillance in West Kazakhstan detected measurable concentrations of toxic elements in freshwater fish while radionuclide activities for ¹³⁷Cs and ⁹⁰Sr remained below regulatory limits [53]. Despite the increasing importance of these production systems, measured radionuclide transfer data remain scarce.

### Nuclear accidents as natural experiments

Major nuclear accidents, particularly those at Chernobyl and Fukushima, have provided unique opportunities to investigate radionuclide transfer under real environmental conditions. These events generated extensive long term datasets that have substantially advanced the development and validation of radionuclide transfer models. Following the Chernobyl accident, approximately 11,000 measurements of total beta activity in milk from Belarus were used to reconstruct the temporal dynamics of ¹³¹I and ¹³⁷Cs using ecological transfer models. Subsequent validation by gamma spectrometry demonstrated the robustness of these models while also highlighting their sensitivity to assumptions regarding radionuclide intake [12].

Long term monitoring of wildlife populations, including wild boars and bears in Fukushima and wild boars in the Chernobyl Exclusion Zone, has enabled the application of nonlinear and mixed-effects models to estimate ecological half-lives and seasonal variations in ¹³⁷Cs concentrations within muscle tissue [54, 55]. These datasets have served as valuable natural experiments for evaluating radionuclide transfer models under contrasting climatic and ecological conditions. Reported ¹³⁷Cs half-lives differed markedly between regions. In wild boars, the effective half-life was approximately 7.3 y in Germany compared with approximately 2.6 y in Japan, whereas the corresponding ecological half-lives were approximately 10.2 y and 2.8 y, respectively [39]. More than a decade after the Fukushima accident, radionuclide concentrations in most food products remain well below regulatory limits; however, persistently elevated ¹³⁷Cs concentrations in wild boars continue to represent the so-called "wild boar paradox" [1, 56].

Despite these advances, important uncertainties remain regarding the extrapolation of radionuclide transfer data from wildlife to livestock, the validity of equilibrium assumptions, and the separation of physical radioactive decay from ecological processes. Transfer coefficients vary substantially among ecosystems and according to the time elapsed since radionuclide deposition, resulting in order-of-magnitude uncertainty in dose assessments. Furthermore, differences between forest ecosystems and agricultural production systems remain insufficiently harmonized, limiting the broader application of existing radionuclide transfer models.

### Uncertainty and transferability of coefficients

Uncertainty in radionuclide transfer to milk and meat is substantial, highly context-dependent, and represents a major limitation to the application of generic Fm and Ff values. Variability among farms reflects differences in soil chemistry, pasture composition, climate, livestock management, and animal physiology. Even within the same nuclear-affected region, transfer of Sr to milk differed markedly among villages [18], whereas Mediterranean and temperate production systems exhibited systematically different transfer patterns for Cs and Sr [57]. Seasonal variation further complicates transfer estimates. In Ukraine, ¹³⁷Cs activity in milk increased from less than 2–88 Bq·L⁻¹ during winter housing to 100–350 Bq·L⁻¹ during the grazing season, with approximately 70% of samples exceeding regulatory limits during peak grazing periods [58]. These observations demonstrate that equilibrium-based transfer coefficients may underestimate the temporal variability associated with changing exposure conditions [19].

Diet composition represents an additional source of uncertainty. Soil-contaminated forage and soluble radionuclide contamination introduced through filtrates produce substantially different Ff values [14, 20], whereas mixed feeding systems alter radionuclide bioavailability and effective transfer to animal products [5, 59]. Reported Fm and Ff values commonly span several orders of magnitude and generally follow lognormal distributions, supporting the application of stochastic methods and Monte Carlo simulations for uncertainty analysis [5, 19]. Furthermore, mechanistic relationships involving the interaction between ⁴⁰K and ¹³⁷Cs, together with Ca- and K-dependent partitioning among milk fractions, provide a biological basis for nutrient-stratified transfer models that incorporate mineral composition and physiological status [21, 22, 26, 60].

Collectively, these findings indicate that radionuclide transfer coefficients should be regarded as context-specific parameters rather than universal constants. Future transfer models should incorporate environmental conditions, dietary composition, seasonal dynamics, and physiological variability to improve predictive accuracy and reduce uncertainty in dose assessments.

The diagram illustrates the hierarchical contributions of environmental contamination, including soil, water, and atmospheric deposition; feed-related factors, including feed source, composition, and radionuclide bioavailability; and animal physiological processes, including gastrointestinal absorption, metabolism, tissue distribution, and lactational secretion, to radionuclide transfer through the food chain.

The progressively narrowing tornado shape represents the cumulative modification of radionuclide transfer as environmental, dietary, and physiological factors interact, ultimately determining radionuclide concentrations in milk and meat, as well as the associated internal radiation dose to consumers (Figure 2).

### Probabilistic modeling and uncertainty quantification

Because radionuclide transfer parameters generally follow lognormal distributions, deterministic point estimates fail to adequately represent the upper tail of exposure distributions that is most relevant for dose assessment. Consequently, probabilistic approaches provide a more realistic framework for quantifying uncertainty. Monte Carlo simulations applied to contaminated meadows in the Narodychi region reported ¹³⁷Cs and ⁹⁰Sr activities in milk and muscle as median values, geometric standard deviations, and 90th percentile confidence bounds, along with the probability of exceeding established hygienic standards [58, 61]. Similarly, compilations of radionuclide transfer data consistently report lognormal rather than fixed transfer coefficients [19], and field measurements have demonstrated that Fm values can vary by up to four orders of magnitude within a single geographical region [18].

These observations support two key recommendations for radionuclide transfer modeling. First, exposure models should propagate uncertainty using stochastic methods, sampling lognormally distributed Fm, Ff, and radionuclide intake values via Monte Carlo simulations to generate credible confidence intervals for predicted radiation doses [5, 19]. Second, sensitivity analyses should identify and rank the principal contributors to model uncertainty, including environmental contamination, feed source and radionuclide bioavailability, and physiological processes governing radionuclide absorption, metabolism, and tissue distribution (Figure 2). Identifying the dominant sources of uncertainty facilitates more efficient data collection and supports the prioritization of intervention strategies and countermeasures [54, 59].

Because radionuclide transfer from feed-to-animal products alone does not determine the activity ultimately consumed by humans, additional modification occurs during food processing and preparation. The following section therefore examines the influence of dairy processing, meat processing, and culinary practices on radionuclide redistribution.

**PROCESSING:** Effects of food processing and culinary practices on radionuclide distribution

### Dairy processing

To our knowledge, this review provides the first integrated evaluation of the effects of food processing on radionuclide redistribution within a One Health framework for risk reduction, directly linking dairy and meat processing with downstream implications for consumer radiation exposure. Dairy processing redistributes radionuclides based on their physicochemical properties, resulting in systematic yet product-specific changes in radionuclide concentrations. Industrial mass-balance studies have demonstrated that lipophilic contaminants preferentially partition into cream and butter [62]. In contrast, fission products such as ¹³⁷Cs and ⁹⁰Sr are predominantly water-soluble and/or associated with protein fractions, resulting in relatively low radionuclide concentrations in cream and butter but higher retention in skim milk and whey fractions [6].

Cheesemaking provides the strongest quantitative evidence for radionuclide redistribution during dairy processing. Fr for ¹³⁷Cs and ⁴⁰K ranging from 0.07 to 0.34 indicate preferential partitioning of these radionuclides into whey, although considerable variation exists among cheese varieties, milk composition, and animal feeding systems [6]. Long term ecological monitoring further indicates that radionuclide concentrations in cheese generally reflect those in the source milk but may increase following seasonal dietary changes, particularly during winter feeding. Concentrations of alpha-emitting radionuclides, including ²²²Rn, ²²⁶Ra, and ²³⁸U, detected in commercial cheeses have generally remained below internationally accepted dose limits [63].

Evidence regarding fermented dairy products remains limited and is based largely on indirect observations. Current findings suggest that fermentation itself is unlikely to cause substantial radionuclide redistribution beyond that resulting from the physical separation of milk fractions; however, quantitative experimental evidence remains insufficient.

### Meat processing and culinary treatment

Meat processing modifies radionuclide distribution through physicochemical partitioning, removal of specific tissues, and mass transfer during food preparation.

Removal of bone and bone marrow reduces exposure to bone-seeking radionuclides such as Sr and Ra. During cooking, heat weakens the bone’s mineral matrix and promotes radionuclide release into cooking liquids. Consequently, bone-in preparations, including soups and smoked fish products, may result in greater dietary radionuclide intake than trimmed meat products. These differences also contribute to methodological uncertainty when studies define edible fractions inconsistently [64].

Comparative studies have shown that boiling and steaming are generally more effective than grilling for removing water-soluble radionuclides such as ¹³⁷Cs. Average residual fractions (Fr) for r-Cs are approximately 0.90 after grilling, 0.60 after boiling, and 0.50 after steaming, corresponding to radionuclide reductions of approximately 11%, 41%, and 47%, respectively [65]. Grilling may increase apparent radionuclide concentrations due to dehydration, whereas boiling promotes radionuclide loss through leaching into the cooking water [66–68]. In seafood, radionuclide retention decreases progressively as boiling time increases, with particularly low residual concentrations reported after 40 min of cooking across several product categories [67]. Nevertheless, the effectiveness of cooking depends on the radionuclide involved, food matrix, cooking conditions, and the water-to-meat ratio, limiting broad generalization across food products. Studies using comparable muscle portions have demonstrated that r-Cs removal closely follows tissue water loss, with reductions in water content negatively correlated with both processing coefficients and residual fractions, indicating that soluble Cs is removed together with tissue water during cooking [65]. Future investigations should therefore report cooking duration, cooking temperature, and water-to-meat ratio to improve reproducibility and facilitate comparison among studies.

Salting may reduce Cs and Sr concentrations but has relatively limited effects on several activation products and transuranic radionuclides. Brining has been shown to reduce Cs concentrations in reindeer meat and beef, whereas the effectiveness of smoking depends on radionuclide binding characteristics and whether bones are retained during processing [65]. Overall, the effectiveness of culinary processing remains radionuclide-specific and highly dependent on processing conditions.

### Processing factors in dose assessment

Food processing introduces systematic, radionuclide-specific, and process-dependent differences between radionuclide concentrations measured in raw animal products and those present in foods at the time of consumption. However, these effects remain inconsistently incorporated into ingestion dose models. Conventional transfer parameters, including Fm, Ff, and CR, describe radionuclide transfer between pasture and animal products under equilibrium conditions [18, 19, 28, 69]. More realistic exposure assessments require additional processing-specific parameters, including the processing coefficient (Pf = C_after/C_before) and the Fr [6, 19].

Comparative studies have demonstrated pronounced radionuclide fractionation during dairy processing. The low Fr reported for ¹³⁷Cs and ⁴⁰K in cheese (0.07–0.34) indicates preferential transfer of these radionuclides into whey during cheesemaking [6]. Conversely, dehydration processes, such as milk powder production, increase radionuclide activity concentrations per unit mass by removing water, thereby increasing the apparent concentrations of ⁴⁰K, ²²⁶Ra, and ²³²Th on a dry-mass basis [18, 27, 70, 71]. Similar concentration effects occur during the production of dried and cured meat products [19]. In addition, the interval between milking or slaughter and consumption allows radioactive decay of short-lived radionuclides, particularly ¹³¹I, thereby reducing Pf values in aged dairy and meat products [19, 72].

Additional uncertainty arises from water loss during processing, redistribution of radionuclides among tissues (e.g., Cs in muscle, Sr in bone, and Am/Pu in bone and liver), and substantial variation in culinary practices among regions and cultures [7, 20]. Failure to account for processing effects may bias ingestion dose estimates, leading to overestimation for radionuclides that partition into whey during cheesemaking and underestimation for dehydrated foods or products containing short-lived radionuclides.

Representative Pf and Fr describing the effects of dairy and meat processing on radionuclide redistribution are summarized in Table 6, based on published experimental studies and food processing investigations [6, 19, 65, 67, 72–77].

**REGULATIONS:** Standards, monitoring frameworks, and analytical quality control

### International regulatory frameworks

Because food processing modifies, but does not eliminate, radionuclides, regulatory limits and systematic monitoring remain the principal safeguards for protecting consumers. International food safety standards provide the basis for national regulatory limits governing ¹³⁷Cs and other radionuclides in milk and meat [78–80]. Comparative monitoring studies conducted in China, Sweden, Serbia, and regions affected by the Semipalatinsk nuclear test site consistently report concentrations of ¹³⁷Cs and ⁹⁰Sr below Codex Alimentarius or more stringent national regulatory limits, with corresponding effective radiation doses considered negligible for consumers [7, 79, 80]. Serbia's post-Chernobyl regulatory limit of 15 Bq·kg⁻¹ for ¹³⁷Cs, which is more stringent than several European Union-derived benchmarks, illustrates differences among national regulatory systems, while long term monitoring demonstrates a substantial decline in radionuclide concentrations over time [78].

Despite broad international agreement, regulatory harmonization remains incomplete. Transuranic radionuclides, including ²⁴¹Am and Pu isotopes, are frequently modeled rather than routinely monitored and are not uniformly incorporated into food safety regulations, creating uncertainty in regulatory compliance and risk interpretation [7]. Furthermore, spatial heterogeneity and localized legacy contamination challenge the assumption of uniform food safety and emphasize the importance of site-specific surveillance programs.

Process control systems, such as Hazard Analysis and Critical Control Points (HACCP), have demonstrably reduced radionuclide and heavy metal contamination throughout meat-processing chains in contaminated regions [81]. Nevertheless, incorporation of radiological criteria into routine food safety audits remains inconsistent among countries and production sectors [78, 82].

**Table 6:** Summary of Pf and Fr describing radionuclide redistribution during dairy and meat processing.

**Process or product**	**Radionuclide(s)**	**Pf** ** or Fr**	**Effect (direction or percentage change)**	**Reference**
Cheesemaking (milk → curd)	¹³⁷Cs, ⁴⁰K	Fr = 0.07–0.34	Indicates that most radionuclide activity is partitioned into the whey fraction	[6]
Butter and cream (milk → fat fraction)	¹³⁷Cs, ⁹⁰Sr	Low Fr (water-soluble radionuclides depleted)	Cs and Sr preferentially partition into skim milk and whey rather than the fat fraction	[73, 74]
Boiling of meat and seafood	¹³⁷Cs, ⁴⁰K	Fr ≈ 0.60	Approximately 41% removal from meat through leaching into broth; approximately 44.6% removal from seafood; retention decreases with prolonged boiling	[65, 67]
Steaming of meat	¹³⁷Cs	Fr ≈ 0.50 (range: 0.40–0.70)	Approximately 47% removal	[65]
Grilling of meat	¹³⁷Cs	Fr ≈ 0.90 (range: 0.70–1.00)	Approximately 11% removal; dehydration may increase apparent radionuclide concentration	[65]
Salting and brining of meat	¹³⁷Cs	Reduction of approximately 50%–86%	Water-soluble Cs is leached into the brine; removal depends on salting protocol	[75, 76]
Soy processing (soybean → soy milk → tofu/yuba)	r-Cs	Approximately 65% transferred to soy milk; 30% retained in okara; tofu and yuba retained 21% and 27%, respectively	Radionuclide concentration in yuba increased approximately 1.7-fold because of water evaporation	[77]
Soy processing (residual pulp)	⁹⁰Sr	Approximately 64% retained in okara	⁹⁰Sr was quantifiable only in the okara fraction	[77]
Storage and aging of foods containing short-lived radionuclides	¹³¹I	Product activity decreases progressively during storage	The physical half-life of ¹³¹I is 8.03 d; delays between production and consumption reduce radionuclide activity in stored foods	[19, 72]

Abbreviations = Fr = Retention factor; Pf = Processing factor; r-Cs = Radiocesium.

### National regulatory models

Although international guidance from the Codex Alimentarius promotes harmonized food safety standards, individual countries set radionuclide limits based on national dietary habits, agricultural production systems, analytical capacity, and risk management policies. In many jurisdictions, regulatory limits are derived from a population radiation dose criterion of ≤1 mSv/year, from which permissible activity concentrations for key radionuclides, including ¹³⁴Cs, ¹³⁷Cs, ⁹⁰Sr, and Pu isotopes, are calculated using dose conversion coefficients and food consumption models [56, 61, 71, 83].

Japan employs a category-specific regulatory framework that incorporates a precautionary screening level equal to one-quarter of the legal limit. For general foods, this corresponds to a screening threshold of 25 Bq·kg⁻¹, with results at or below this value routinely reported as "not detected" [1]. Although this approach provides an additional safety margin, it may also create ambiguity because the designation "not detected" reflects an administrative reporting threshold rather than the analytical LOD or LOQ.

Following the Chernobyl accident, regulatory limits adopted within the European Union and Serbia for ¹³¹I and the combined activity of ¹³⁴Cs and ¹³⁷Cs in milk and other foods generally ranged from several hundred to a few thousand Bq·kg⁻¹ (or Bq·L⁻¹), whereas studies conducted in Kazakhstan have evaluated radionuclide concentrations in milk and meat relative to permissible levels established for regions influenced by the Semipalatinsk Test Site [7, 78].

Long term regional monitoring programs, including those implemented in the Narodychi region of Ukraine, integrate probabilistic soil–grass–milk transfer models with regulatory intervention thresholds, such as the PL-2006 framework, to establish area-specific criteria for pasture management, agricultural restrictions, and environmental remediation [61]. These programs illustrate the increasing integration of predictive transfer modeling into evidence-based regulatory decision-making.

Representative national and international regulatory limits for radionuclides in milk and meat are summarized in Table 7, based on current international guidance, national regulations, and long term monitoring programs [1, 2, 7, 8, 56, 61, 83–85].

Key sources of uncertainty arise from differences among countries in analytical detection and reporting conventions, radionuclides included within regulatory frameworks (particularly transuranic radionuclides), and assumptions regarding food consumption embedded in dose assessment models. Nevertheless, enforcement data consistently demonstrate high compliance despite differing regulatory limits. In Japan, r-Cs concentrations in foods remain substantially below regulatory limits [1, 56]. Similarly, in the United Kingdom, only 0.0013% of more than one million analyzed food samples exceeded regulatory limits, contributing to the removal of import controls in 2024 [85]. Australia and New Zealand continue to apply Codex Alimentarius guideline levels, with milk serving as a sentinel food for environmental surveillance [84]. However, important knowledge gaps remain for transuranic radionuclides and NORM, which are more frequently estimated through modeling than measured directly [7, 8].

**Table 7:** Representative national and international regulatory limits for radionuclides in milk and meat.

**Jurisdiction or standard**	**Product**	**Radionuclide(s) or regulatory basis**	**Regulatory limit or trigger**
Japan (post-Fukushima)	General foods	Sum of ¹³⁴Cs + ¹³⁷Cs	100 Bq·kg⁻¹ (regulatory limit) [56]
Japan	Milk and infant foods	¹³⁴Cs + ¹³⁷Cs	50 Bq·kg⁻¹ [1, 56]
Japan	Drinking water	¹³⁴Cs + ¹³⁷Cs	10 Bq·L⁻¹ [56]
Japan	Dose-based framework	All food radionuclides	Design target ≤1 mSv/year from food consumption [56]
European Union	Milk and milk products	¹³⁷Cs	370 Bq·kg⁻¹ [2, 83]
European Union	Other food products	¹³⁷Cs	600 Bq·kg⁻¹ [2, 83]
Poland	All food products	Radionuclides with half-lives >10 d, primarily ¹³⁴Cs and ¹³⁷Cs	1250 Bq·kg⁻¹ [2]
Ukraine (PL-2006)	Milk and cattle muscle (meat)	¹³⁷Cs and ⁹⁰Sr	Product-specific MPLs [61]
Kazakhstan (Semipalatinsk region)	Cow milk	¹³⁷Cs / ⁹⁰Sr	100 / 25 Bq·L⁻¹ [7]
Kazakhstan (Semipalatinsk region)	Meat (cattle, sheep, goats, and horses)	¹³⁷Cs / ⁹⁰Sr	200 / 50 Bq·kg⁻¹ [7]
New Zealand	Milk	¹³⁷Cs, ⁹⁰Sr, and ¹³¹I	Anthropogenic radionuclide concentrations remain well below Codex guideline levels; milk is used as a sentinel food for surveillance [84]
United Kingdom (Commission Implementing Regulation (EU) 2016/6)	Imported foods	Cs	100 Bq·kg⁻¹; only 0.0013% of 1,485 analyzed samples exceeded the limit [85]
IAEA guidance	Foods (general)	²¹⁰Pb, ²³⁴U, ²³⁸U, ²²⁶Ra, and ²²⁸Ra	0.1–1 Bq·kg⁻¹ for Ra and Pb radionuclides; 10 Bq·kg⁻¹ for U radionuclides [8]

Abbreviations = Cs = Cesium; IAEA = International Atomic Energy Agency; MPL = Maximum permissible level.

### LOD, LOQ, and measurement uncertainty

High-purity germanium (HPGe) gamma spectrometry remains the reference analytical method for determining ¹³⁴Cs, ¹³⁷Cs, and ⁴⁰K in milk and meat. However, reported LODs and minimum detectable activities (MDAs) vary by more than one order of magnitude depending on counting time, sample matrix, detector characteristics, and the statistical criteria used for calculation [2, 56, 86]. Several technical reports define the LOD as the activity corresponding to a relative standard deviation (RSD) of 30% and the LOQ as that corresponding to an RSD of 10%. Under a counting time of 43,200 s, reported values include an LOD of 0.16 Bq·kg⁻¹ and an LOQ of 0.48 Bq·kg⁻¹ for ¹⁵²Eu, together with MDA values of 1.34, 1.83, and 4.39 Bq·kg⁻¹ for ²²⁶Ra, ²²⁸Ra, and ⁴⁰K, respectively [87, 88]. Such methodological differences reduce comparability among laboratories and countries.

Several studies evaluating ⁹⁰Sr have applied the Currie approach for calculating LOD and LOQ, reporting an LOD of 0.011 Bq·kg⁻¹ and an LOQ of 0.024 Bq·kg⁻¹. These methods were validated using certified reference materials and interlaboratory proficiency testing, supporting their analytical reliability [89]. Achieving comparable regulatory decisions therefore requires harmonized quality assurance and quality control (QA/QC) procedures, including standardized Currie-based LOD and LOQ calculations, ISO/IEC 17025 accreditation, routine use of certified reference materials, and participation in proficiency testing schemes [81, 89].

### Analytical methods and quality assurance

Reliable quantification of radionuclides in milk and meat depends on complementary radiochemical and spectrometric methods whose analytical performance underpins regulatory confidence. High-purity germanium (HPGe) gamma spectrometry remains the reference technique for determining ¹³⁷Cs and many naturally occurring radionuclides in national monitoring programs and has consistently demonstrated compliance with regulatory limits since the Chernobyl accident [78, 90, 91]. In contrast, determination of ⁹⁰Sr requires radiochemical separation followed by beta counting, where chemical recovery, separation efficiency, and yield correction constitute the principal sources of analytical uncertainty [89, 92, 93]. Comparative investigations have shown that matrix effects in samples with high fat or protein concentrations can bias radionuclide measurements unless validated microwave ashing or wet-digestion procedures are employed.

A continuing challenge concerns the distinction between analytical detection capability and regulatory decision thresholds. Parameters such as MDA, chemical recovery, counting time, and detector efficiency may influence whether samples close to regulatory limits are classified as compliant or non-compliant. Consequently, analytical methods should be validated in accordance with ISO/IEC 17025 requirements, including assessment of specificity, linearity, recovery, precision, measurement uncertainty, and MDA, to ensure comparability among laboratories and provide legally defensible results [81, 89].

Cross-system variability also reflects differences in laboratory infrastructure, calibration procedures, reference standards, and implementation of QA/QC programs across countries. Integration of radionuclide monitoring into HACCP systems, in which ¹³⁷Cs and ⁹⁰Sr are managed as chemical hazards with defined critical limits, validated analytical methods, and accredited verification procedures, further strengthens food safety governance and enhances confidence in regulatory compliance [81].

**COUNTERMEASURES:** Evidence-based strategies for risk reduction

### Farm-level interventions

Current evidence consistently supports integrated farm-level countermeasures to reduce radionuclide transfer to milk and meat, although their effectiveness varies with radionuclide characteristics, animal species, production systems, and environmental conditions. Restricting grazing on contaminated pastures and replacing contaminated forage with low-contamination conserved feeds remain among the most effective interventions because feed represents the principal pathway for radionuclide intake in livestock [7, 59, 61, 86, 94]. Similarly, "clean feeding" for several weeks exploits the biological elimination of radionuclides and substantially decreases ¹³⁷Cs concentrations in milk and meat. In contrast, reductions in ⁹⁰Sr occur more slowly because of its preferential accumulation in bone and continued uptake through alternative pathways, including drinking water in aquatic production systems [52].

Mineral amendments, including potassium (K), Ca, phosphorus (P), and marl, reduce the absorption of Cs and Sr through competitive ion-exchange mechanisms. These amendments have been associated with significant reductions in ¹³⁷Cs concentrations in animal products while simultaneously improving soil fertility and animal health under acidic, Cs-contaminated conditions [95, 96]. Gastrointestinal sorbents, including ferrocyanides, clay minerals, pectin, and phytogenic formulations, further accelerate radionuclide elimination, reducing ¹³⁷Cs and ⁹⁰Sr concentrations toward permissible limits and shortening biological half-lives to approximately 15 d [5, 97].

Management of soil and feed resources remains fundamental for long term risk reduction. Elevated soil concentrations of ¹³⁷Cs have been associated with increased Pb, Cd, and radionuclide accumulation in animal products [98], whereas K fertilization has reduced plant uptake of ¹³⁷Cs by approximately 2.7- to 2.9-fold [95]. Radioprotective feed formulations containing phytogenic ingredients, such as sea buckthorn (*Hippophae* *rhamnoides*), Jerusalem artichoke (*Helianthus tuberosus*), and wormwood (*Artemisia absinthium*), combined with pectin and/or ferrocyanide, have also been shown to reduce radionuclide concentrations in meat and restore the ¹³⁷Cs/⁹⁰Sr ratio to acceptable levels [97]. Available evidence indicates that the greatest reductions are achieved by combining plant-derived additives, selective sorbents, and functional mineral premixes that enhance gastrointestinal radionuclide binding and excretion [99, 100].

Food preparation can further reduce radionuclide concentrations. Prolonged boiling and stewing of meat from contaminated regions significantly decrease ¹³⁷Cs and ⁹⁰Sr concentrations because these radionuclides are partially water-soluble and redistribute into cooking liquids during thermal processing [101]. Overall, reduction of radionuclide concentrations in milk and meat is achieved primarily through minimizing feed contamination, applying mineral amendments and selective sorbents, and optimizing soil and manure management to produce uncontaminated feed. Food processing should therefore be regarded as a complementary intervention that further decreases residual radionuclide concentrations before consumption.

### Supply chain level measures

Supply chain interventions complement farm-level countermeasures but require implementation strategies tailored to local contamination patterns and production systems. Spatial zoning based on radionuclide deposition and the soil–plant–animal transfer pathway is consistently recommended for managing contaminated agricultural areas [102]. Public monitoring databases further improve transparency, facilitate regulatory oversight, and strengthen public confidence in food safety systems [78]. In addition, digital traceability technologies, including radio-frequency identification, improve batch-level segregation, product tracking, and recall efficiency. However, harmonized monitoring indicators and interoperability of cross-border surveillance systems remain underdeveloped [103].

Processing-based radionuclide redistribution also contributes to supply chain risk management but is radionuclide dependent. During cheesemaking, ¹³⁷Cs and ⁴⁰K preferentially partition into whey, resulting in relatively low retention in the cheese curd and allowing moderately contaminated milk to be diverted into processing streams that reduce radionuclide concentrations in finished products [6]. Likewise, thermal processing of meat decreases ¹³⁷Cs and ⁹⁰Sr concentrations to varying degrees and may complement product segregation and blending strategies [101]. Nevertheless, debate continues regarding whether radionuclide redistribution during processing represents true risk reduction or merely transfers radioactivity into processing by-products that require appropriate disposal.

Operational measures such as directing milk with relatively high ¹³⁷Cs concentrations to butter and cream production, sourcing fresh meat from less contaminated regions, and implementing HACCP systems with radionuclide-specific critical limits have demonstrated effectiveness when integrated with existing regulatory frameworks [78, 81, 102]. Uncertainty nevertheless remains due to heterogeneous contamination, radioactive decay over time, and the management of contaminated by-products. From an economic perspective, administrative interventions, including zoning, product segregation, diversion, and traceability, generally represent the most cost-effective first-line measures. In contrast, long term use of ammonium ferric hexacyanoferrate (AFCF; Prussian blue) boluses and sorbent supplementation incurs recurring per-animal costs that are justified primarily under conditions of persistent or severe contamination [104, 105].

### Consumer-level measures

Risk communication is most effective when recommendations are behavior-oriented, tailored to specific audiences, and communicated through trusted information sources [106–108]. However, uncertainty remains about the relative effectiveness of narrative versus statistical communication approaches, and increasing reliance on social media has raised additional concerns about the credibility of health information. Furthermore, substantial demographic differences in risk perception limit the effectiveness of universal communication strategies, highlighting the need for standardized methods to evaluate communication outcomes and strengthen collaboration among public health, agricultural, and regulatory sectors.

Targeted nutritional interventions do not directly reduce radionuclide body burdens but may improve physiological resilience and reduce susceptibility to adverse health outcomes associated with environmental stressors. Consequently, the potential contribution of nutritional resilience to mitigating health impacts under chronic radiological exposure warrants further investigation.

Representative evidence describing the effectiveness, duration, feasibility, and relative cost of farm-, supply chain-, and consumer-level countermeasures for reducing radionuclide concentrations in milk and meat is summarized in Table 8, based on published experimental studies and field evaluations [6, 52, 65, 95–97, 104–106, 109–113].

## KNOWLEDGE GAPS AND RESEARCH PRIORITIES

### Data limitations and modeling challenges

Despite the availability of several effective countermeasures, major knowledge gaps hinder the optimal selection and implementation of these countermeasures. Comparative evidence reveals substantial data fragmentation, regional bias, and methodological heterogeneity, all of which limit the robustness and transferability of radionuclide transfer models. Comprehensive datasets for major fission products, particularly ¹³⁷Cs and ⁹⁰Sr, remain incomplete, whereas transuranic radionuclides and minor livestock species are underrepresented or assessed using surrogate transfer values [5, 7, 14, 25, 59]. IAEA compilations are dominated by temperate production systems, with limited representation of tropical and Mediterranean agroecosystems, thereby restricting extrapolation across climates and farming systems [18, 57]. Reported Sr Fm values span approximately four orders of magnitude, further illustrating the magnitude of parameter uncertainty [18]. The use of stable Cs and Sr analogs may partly overcome analytical constraints associated with low radionuclide activity; however, correlations between stable elements and their radioactive counterparts remain incomplete, particularly for Sr [57, 114].

Many existing models oversimplify physiological determinants such as age, productivity, and soil ingestion and fail to account adequately for scenario-specific radionuclide bioavailability. This limitation is important because soil-bound radionuclides may exhibit transfer rates several orders of magnitude lower than those associated with soluble or feed-borne contamination [7, 20]. Transfer coefficients themselves may vary by three to four orders of magnitude, rendering deterministic dose projections highly unstable [18]. Although mechanistic and multicompartment modeling approaches have been developed, their validation across animal species, production systems, and climatic regions remains limited [59].

**Table 8:** Effectiveness, duration, feasibility, and relative cost of countermeasures for reducing radionuclide concentrations in milk and meat at farm, supply chain, and consumer levels.

**Countermeasure (level)**	**Target radionuclide(s)**	**Effectiveness (reduction)**	**Duration or onset**	**Cost and feasibility**	**Reference**
Clean feeding or substitution with uncontaminated fodder (farm; fish production)	¹³⁷Cs, ⁹⁰Sr	Up to approximately 10-fold reduction in milk and meat; generally 2- to 5-fold reduction	Rapid response following contamination; greatest benefit during the first year	Highly cost-effective	[52, 105, 109]
Hexacyanoferrate (Prussian blue/ferrocyanide) powder, 3-5 g/cow/day (farm)	¹³⁷Cs	Up to 90% reduction in milk	Requires continuous daily administration	Inexpensive; effective Cs-binding agent	[104, 110, 111]
Hexacyanoferrate rumen bolus, 30 g single dose (farm)	¹³⁷Cs	50%-75% reduction	Approximately 2 months per administration	Single treatment; easy implementation	[111]
Hexacyanoferrate salt licks or Bifege® (farm)	¹³⁷Cs	Approximately two-fold reduction with salt licks; 90%-95% reduction with Bifege®	Approximately 10 d	Low cost	[111]
Clay binders (bentonite, Bolus alba) (farm)	Cs	Effective but approximately 88-266 times less effective than AFCF	Not specified	May reduce mineral and trace-element availability; logistical limitations	[112]
Mineral amendments (K fertilizers, combined P + K fertilization, liming) (soil)	¹³⁷Cs	K fertilization reduces plant uptake by approximately 2.7- to 2.9-fold	Approximately 3 years	Cost-effectiveness not quantified	[95, 96, 113]
Grazing land and pasture management (soil)	¹³⁷Cs	Approximately 2.8-fold reduction in pasture uptake	One-time intervention with multi-year effect	High initial cost; declining long term benefit	[113]
Phytogenic radioprotective premixes with pectin and ferrocyanide (farm)	¹³⁷Cs, ⁹⁰Sr	Approximately 15% reduction in meat; normalization of the ¹³⁷Cs/⁹⁰Sr ratio	Feeding period of approximately 3-4 months	Potentially sustainable; locally sourced	[97]
Culinary processing (consumer)	¹³⁷Cs	Boiling: approximately 41% reduction; steaming: approximately 47% reduction	During meal preparation	Negligible cost	[65]
Cheese diversion or processing-route selection (supply chain)	¹³⁷Cs, ⁴⁰K	Fr = 0.07-0.34 during cheesemaking	During dairy processing	Low cost; utilizes existing processing infrastructure	[6]
Dietary advice and risk communication (consumer)	All radionuclides	Variable reduction in dietary intake	Immediate	Very low cost per unit of avoided radiation dose	[104, 106]

Abbreviations = AFCF = Ammonium ferric hexacyanoferrate; Fr = Retention factor; K = Potassium; P = Phosphorus.

### Understudied radionuclides and NORM contexts

Monitoring and modeling remain disproportionately focused on ¹³⁷Cs and ⁹⁰Sr. In contrast, transuranic radionuclides, including ²⁴¹Am and ²³⁹⁺²⁴⁰Pu, are rarely measured directly in milk and meat. Available estimates are predominantly model-based, predict very low activity concentrations, and lack harmonized validation and systematic market-level surveillance [7]. Beyond the U–Th–Ra decay series, radionuclides such as ²¹⁰Pb and ²²⁶Ra have been detected in food systems, but livestock-specific datasets remain sparse and vary substantially among countries [8, 79]. Long term datasets integrating NORM with anthropogenic radionuclides are largely restricted to regions affected by nuclear accidents, limiting broader comparisons among production systems [12, 115, 116].

An additional unresolved issue concerns combined exposure to radionuclides and other environmental contaminants. Most studies report single-radionuclide activities and calculate independent dose estimates despite the frequent co-occurrence of radionuclides with toxic elements, including Pb and Cd alongside ¹³⁷Cs [98, 101]. Potential interactions among contaminants and the propagation of uncertainty under mixture exposure are rarely quantified. Dietary pathways also differ substantially among regions. In some areas affected by the Chernobyl accident, internal exposure to ¹³⁷Cs is driven more strongly by forest-derived foods than by milk or meat [117]. This finding further demonstrates that the relative importance of animal products is highly dependent on local dietary practices and environmental conditions.

### Food processing and real-world dietary patterns

Exposure assessment is further constrained by incomplete characterization of food processing factors and actual dietary behaviors. Fermented foods produced in East Africa and Southeast Asia exhibit substantial compositional and microbiological variation, while processing conditions are often reported inconsistently [118–120]. Consequently, available evidence is insufficient to define robust and transferable Pf and Fr values for many traditional and fermented animal-derived foods.

Dietary databases also frequently fail to distinguish adequately among processing intensity, preparation methods, and final consumed forms. Misclassification within systems such as NOVA and ultra-processed food categories may further obscure the relationship between measured radionuclide concentrations in raw commodities and actual consumer exposure. Few dose assessment studies incorporate variability in cooking, fermentation, storage, dehydration, or fractionation into probabilistic exposure models. This omission may lead to systematic overestimation or underestimation of ingestion doses, depending on the radionuclide and processing method.

### Need for baseline monitoring data for milk and meat

Baseline monitoring is widely recognized as a central component of radiological preparedness, but implementation remains inconsistent among countries and regions. Evidence from Germany, Texas, Chernobyl-affected regions, and Japan demonstrates that pre-incident reference datasets allow statistically defensible attribution of contamination and improve comparisons between background and post-accident radiation doses [1, 40, 98, 121–123]. However, differences in feeding systems, soil characteristics, climate, animal species, and production practices limit the transferability of baseline values across regions.

Experience from the Chernobyl and Fukushima accidents emphasizes the importance of predefined sampling frameworks that specify which commodities should be sampled, where sampling should occur, and how frequently samples should be collected [12, 122]. Power-based sample size calculations and prioritization of widely consumed sentinel commodities, particularly milk, are also essential. Persistent challenges include differences in analytical methods, detection capabilities, reporting thresholds, and laboratory quality assurance. High-throughput analytical protocols, including methods developed for ⁹⁰Sr determination in milk, may improve consistency and facilitate rapid emergency monitoring [124]. Nevertheless, quantitative uncertainty in baseline measurements remains poorly reported, particularly with regard to temporal variability and its propagation into emergency dose models.

**Research roadmap:** Priorities and actionable recommendations

Based on the identified evidence gaps, a prioritized research and governance agenda is required. First, well-designed multispecies transfer studies should be conducted in tropical, Mediterranean, and low-resource production systems and should include underrepresented livestock and aquatic species, such as goats, sheep, buffaloes, camels, and farmed fish. These studies should generate directly measured values of Fm and Ff under clearly defined environmental, dietary, and physiological conditions.

Second, physiologically based pharmacokinetic and multicompartment models should be developed and cross-validated across species, climates, and production systems. Such models should incorporate age, productivity, tissue partitioning, soil ingestion, dietary composition, and radionuclide bioavailability.

Third, probabilistic uncertainty analysis should become the default modeling approach. Transfer coefficients and intake parameters should be represented using appropriate probability distributions, particularly lognormal distributions, and assessed through Monte Carlo simulation and global sensitivity analysis.

Fourth, future studies should examine combined exposure to radionuclides, toxic elements, and climate-related stressors. This includes evaluating how drought, flooding, soil acidification, changing forage composition, and altered water availability influence contaminant mobility and transfer.

Fifth, artificial intelligence and machine learning methods should be evaluated for predictive surveillance, spatial risk mapping, anomaly detection, and sampling prioritization. These approaches should complement, rather than replace, validated analytical measurements and mechanistic understanding.

Sixth, an open-access international database should be established for transfer and processing parameters, including Fm, Ff, CR, Pf, and Fr. Records should include metadata on animal species, age, production system, diet, soil properties, climate, exposure duration, analytical method, and uncertainty.

Finally, internationally harmonized baseline monitoring protocols should be developed, with milk used as a sentinel commodity where appropriate. These protocols should define target radionuclides, sampling frequency, geographical coverage, analytical performance criteria, minimum sample sizes, and procedures for uncertainty reporting. Veterinary-led surveillance platforms integrating animal health, food safety, environmental monitoring, and public health data would strengthen One Health preparedness and support faster, evidence-based responses to future radiological events.

## RADIOLOGICAL SAFETY OF MILK AND MEAT IN THE CONTEXT OF THE ONE HEALTH CONCEPT

Within the One Health framework, the radiological safety of milk and meat is determined by the interconnected environment–animal–human pathway. Radionuclide transfer is highly element-specific. ¹³⁷Cs preferentially accumulates in muscle and is readily transferred to milk, whereas ⁹⁰Sr is primarily deposited in bone and generally contributes less to edible soft tissues. ¹³¹I can appear rapidly in milk after exposure because of its uptake by the thyroid and subsequent secretion, whereas transuranic radionuclides generally exhibit limited transfer to milk and meat. However, transfer coefficients vary according to animal species, diet, soil characteristics, physiological status, and production system, requiring context-specific assessment rather than reliance on universal values.

Food processing further modifies radionuclide distribution. Cheesemaking reduces the retention of many water-soluble radionuclides in curd because a substantial proportion is transferred to whey, whereas dehydration concentrates radionuclide activity per unit mass. Boiling and related moist-heat treatments promote leaching of water-soluble radionuclides from meat into cooking liquids. These effects should be incorporated into exposure assessment because the radionuclide activity present in raw products may differ substantially from that in foods as consumed.

Regulatory systems are generally anchored to dose-based protection criteria, commonly an annual effective dose of ≤1 mSv from food consumption, but activity limits and reporting conventions differ among countries. Effective risk reduction, therefore, requires coordinated interventions at the farm, supply chain, and consumer levels. These include feed and pasture management, mineral supplementation, selective sorbents, processing controls, accredited surveillance, traceability, and risk communication. Their integration within a unified One Health framework strengthens prevention, monitoring, and response across environmental, veterinary, food safety, and public health sectors (Table 9).

**Table 9:** One Health synthesis of the environment–animal–human pathway for radionuclides in milk and meat, including selected findings from 2020–2026 and major intervention points.

**One Health pillar**	**Key processes and findings from 2020–2026**	**Representative findings or examples**	**Intervention and control points**
Environment (soil–plant–deposition)	Atmospheric deposition and soil properties, including clay content, exchangeable K, and organic matter, govern radionuclide bioavailability; soil-to-forage transfer varies by several orders of magnitude	Soil-to-forage transfer differs markedly among production systems; elevated K suppresses r-Cs uptake, whereas clay minerals and humic substances immobilize r-Cs and Ra	K and Ca fertilization; liming; deposition-based zoning; pasture remediation; sourcing feed from uncontaminated land
Animal (livestock physiology and transfer)	Radionuclide transfer is modified by species, diet, age, productivity, and tissue partitioning	Feed-to-milk transfer of r-Cs may be greater in sheep than in cattle; radioiodine transfer to sheep milk is comparatively high; radiostrontium accumulates primarily in bone; biological half-life is generally shorter in milk than in muscle	Clean feeding; grazing restrictions; mineral amendments; gastrointestinal sorbents, including ferrocyanides, clays, and pectin
Human (diet and internal dose)	Milk and meat represent important ingestion pathways; food processing redistributes radionuclide activity; governance is generally based on annual effective dose criteria	R-Cs concentrations in foods from Japan remain well below regulatory limits; almost all monitored United Kingdom import samples complied with regulatory requirements; dietary exposure in New Zealand remains below Codex guideline levels; cheesemaking transfers much of the activity to whey, whereas boiling removes a substantial fraction from meat	Processing and culinary controls; regulatory limits and surveillance; dietary guidance; audience-specific risk communication
Cross-cutting surveillance	Integrated environmental, veterinary, and food monitoring; incorporation of radiological criteria into HACCP systems; establishment of pre-incident reference data	R-Cs and radiostrontium can be managed as critical hazards within HACCP systems; milk serves as a sentinel matrix; baseline datasets improve attribution and post-incident comparisons	Intersectoral One Health surveillance; harmonized sampling and analytical protocols; accredited laboratories; transparent, open-access monitoring databases

Abbreviations = Ca = Calcium; HACCP = Hazard Analysis and Critical Control Points; K = Potassium; Ra = Radium.

## CONCLUSION

This review provides a comprehensive synthesis of current evidence on radionuclide transfer to milk and meat within a One Health framework, integrating environmental contamination, animal physiology, food processing, regulatory oversight, and public health protection. Across the literature, radionuclide transfer was shown to be highly radionuclide-specific and strongly influenced by animal species, production system, diet, soil characteristics, climatic conditions, and physiological status. Among the radionuclides evaluated, ¹³⁷Cs consistently exhibited the greatest transfer to edible tissues, particularly muscle and milk, whereas ⁹⁰Sr accumulated predominantly in bone with comparatively limited transfer to edible products. Short-lived radionuclides such as ¹³¹I rapidly appeared in milk following exposure, while transuranic radionuclides generally displayed minimal transfer to milk and meat. Reported transfer parameters (Fm, Ff, CR, and Tag) varied by several orders of magnitude, emphasizing that radionuclide transfer cannot be represented adequately by universal coefficients and instead requires scenario-specific evaluation. Evidence further demonstrated that food processing substantially modifies radionuclide distribution. Cheesemaking preferentially partitions water-soluble radionuclides into whey, whereas boiling, steaming, and brining reduce radionuclide concentrations in meat through leaching, while dehydration increases activity concentrations on a mass basis. Collectively, these findings confirm that realistic exposure assessment must integrate transfer processes, food processing, and consumer practices rather than relying solely on environmental contamination or feed-to-animal transfer coefficients.

From a practical perspective, the review highlights that effective radiological protection of the food chain requires integrated interventions operating across the entire environment–animal–human continuum. Farm-level measures, including clean feeding, grazing restriction, mineral amendments, selective sorbents, and optimized pasture management, consistently reduced radionuclide transfer to animal products. Supply chain interventions, including product segregation, processing-based redistribution, traceability systems, and HACCP-based monitoring, further strengthened food safety, while consumer-level measures, including evidence-based risk communication and appropriate culinary processing, provided additional reductions in dietary exposure. These complementary strategies support implementation of a One Health approach in which veterinary medicine, environmental science, agriculture, food safety, and public health operate as an integrated system for radiological preparedness and response.

A major strength of this review is its integration of evidence from environmental radioecology, veterinary science, food processing, analytical chemistry, regulatory science, and risk assessment into a single One Health framework. Unlike previous reviews that focused primarily on radionuclide transfer or environmental contamination, this review links radionuclide behavior from environmental deposition through livestock production and food processing to consumer exposure and regulatory management. It also synthesizes recent evidence (2020–2026), compares the strengths and limitations of different transfer metrics (CR, Tag, Fm, and Ff), evaluates probabilistic approaches for uncertainty analysis, and incorporates processing factors (Pf and Fr), regulatory frameworks, quality assurance, and evidence-based countermeasures into a unified conceptual model.

Nevertheless, several limitations remain. Available evidence is dominated by studies of ¹³⁷Cs and ⁹⁰Sr conducted in temperate regions following major nuclear accidents, whereas data for tropical and subtropical production systems, NORM, transuranic radionuclides, and underrepresented livestock species remain limited. Considerable heterogeneity in analytical methods, monitoring strategies, reporting conventions, and transfer models restricts direct comparison among studies. Furthermore, many published transfer coefficients are derived under equilibrium assumptions that may not adequately represent seasonal variation, heterogeneous contamination, changing feeding practices, or evolving environmental conditions. Information on food-processing effects, consumer dietary behavior, and combined exposure to radionuclides and other environmental contaminants also remains insufficient for comprehensive risk assessment.

Future research should prioritize the generation of standardized, experimentally measured transfer data across diverse livestock species and production systems, particularly in tropical and low-resource regions. Development and international validation of physiologically based pharmacokinetic and multicompartment models should be accompanied by routine application of probabilistic uncertainty analyses using Monte Carlo simulation and sensitivity analysis. Greater attention should also be directed toward transuranic radionuclides, NORM, radionuclide–heavy metal interactions, climate-driven changes in contaminant mobility, and processing-specific TF. Establishment of internationally harmonized, open-access databases for Fm, Ff, CR, Tag, Pf, and Fr values, together with standardized baseline monitoring protocols and integrated veterinary-led surveillance systems, would substantially improve preparedness for future radiological emergencies. Emerging technologies, including artificial intelligence, machine learning, digital traceability, and real-time environmental monitoring, also offer promising opportunities to strengthen predictive surveillance and evidence-based decision-making.

In conclusion, radiological safety of milk and meat cannot be evaluated solely from environmental contamination or radionuclide transfer coefficients but must be considered within an integrated One Health framework that encompasses environmental processes, livestock physiology, food processing, regulatory oversight, and consumer exposure. Adoption of harmonized monitoring systems, probabilistic risk assessment, validated analytical methods, and coordinated interventions across the food production chain will improve the accuracy of exposure assessment and strengthen public health protection. As environmental pressures, nuclear technologies, and food production systems continue to evolve, implementation of multidisciplinary, evidence-based One Health strategies will remain essential for ensuring the long term radiological safety of animal-derived foods and safeguarding both animal and human health.

## DATA AVAILABILITY

The data generated during the study are included in the manuscript.

## GENERATIVE AI DECLARATION

The authors declare that generative artificial intelligence (AI) tools were used solely to improve language, grammar, and readability during manuscript preparation. All scientific content and conclusions were developed and verified by the authors. The authors take full responsibility for the accuracy, integrity, and originality of the work presented, and no AI tool was listed as an author.

## AUTHORS’ CONTRIBUTIONS

AS, DS, and SA: Conceptualization, literature search, writing – original draft, and supervision. AK, AZ, and KA: Literature search and writing – original draft. SS, AA, and BA: Literature analysis, visualization, and writing – review and editing. SZ, PA, and VZ: Writing – review and editing and critical revision. AS, SA, and AK: Supervision and writing – review and editing. All authors have read and approved the final version of the manuscript.
